# Evaluation of Acute 13-Week Subchronic Toxicity and Genotoxicity of the Powdered Root of Tongkat Ali (*Eurycoma longifolia* Jack)

**DOI:** 10.1155/2013/102987

**Published:** 2013-08-25

**Authors:** Ching-Hao Li, Jiunn-Wang Liao, Po-Lin Liao, Wei-Kuang Huang, Ling-Shan Tse, Cheng-Hui Lin, Jaw-Jou Kang, Yu-Wen Cheng

**Affiliations:** ^1^Department of Physiology, School of Medicine, College of Medicine, Taipei Medical University, Taipei, Taiwan; ^2^Graduate Institute of Medical Sciences, College of Medicine, Taipei Medical University, Taipei, Taiwan; ^3^Graduate Institute of Veterinary Pathology, College of Veterinary Medicine, National Chung Hsing University, Taichung, Taiwan; ^4^School of Pharmacy, Taipei Medicine University, 250 Wu-Hsing Street, Taipei 11031, Taiwan; ^5^Institute of Toxicology, College of Medicine, National Taiwan University, 1 Jen-Ai Road, Section 1, Taipei 10051, Taiwan

## Abstract

Tongkat Ali (*Eurycoma longifolia*) is an indigenous traditional herb in Southern Asia. Its powdered root has been processed to produce health supplements, but no detailed toxicology report is available. In this study, neither mutagenicity nor clastogenicity was noted, and acute oral LD_50_ was more than 6 g/kg b.w. After 4-week subacute and 13-week subchronic exposure paradigms (0, 0.6, 1.2, and 2 g/kg b.w./day), adverse effects attributable to test compound were not observed with respect to body weight, hematology, serum biochemistry, urinalysis, macropathology, or histopathology. However, the treatment significantly reduced prothrombin time, partial thromboplastin time, blood urea nitrogen, creatinine, aspartate aminotransferase, creatine phosphate kinase, lactate dehydrogenase, and cholesterol levels, especially in males (*P* < 0.05). These changes were judged as pharmacological effects, and they are beneficial to health. The calculated acceptable daily intake (ADI) was up to 1.2 g/adult/day. This information will be useful for product development and safety management.

## 1. Introduction

Herbal medicines, originating in China, Europe, and India, have a rich history. Over the last 30 years, the use of herbal medicines as complementary/alternative therapies has become increasingly popular throughout the world. A study in the *Journal of the American Medical Association* demonstrated that 40% of US residents regularly use these therapies. These people are well educated and are not dissatisfied with conventional medicines [1]. 

*Eurycoma longifolia* Jack (genus: *Eurycoma*; family: Simaroubaceae) is one of the most popular tropical herbal plants in Southeast Asia countries. In these countries, *E. longifolia* is identified locally as “Tongkat Ali” or “Malaysia ginseng” (Malaysia), “Pasak Bumi” or “Bedara Pahit” (Indonesia), “Ian-don” (Thailand), and “Cay ba binh” (Vietnam). It is an evergreen, slow-growing shrub with maximum height of 15–18 m, and it is commonly found in tropical forests. After 2-3 years of cultivation, this dioecious plant, with male and female flowers produced in large panicles on different trees, becomes mature and bears fruit. For commercial uses, their roots are harvested after 4 years of cultivation [2, 3]. Their roots, stems, and bark are used as folk medicines in different ways for the alleviation of various illnesses, including aches, intermittent fever, sexual insufficiency, dysentery, glandular swelling, and fatigue. Traditionally, the water decoction of *E. longifolia *root is consumed. Nowadays, more convenient formulas are available, primarily additives mixed with teas and coffees, and over 200 products are available either in the form of raw crude root powder or as capsules mixed with other herbs in the health food market [3]. Some of these products, such as Libidus and Maxidus (which claim to use *E. longifolia* as the principal ingredient), have been issued with warnings or even banned in the USA, Canada, and Singapore, because of the lack of science-based safety information, the presence of undeclared prescription drugs, and the use of fake ingredients. 

Tongkat Ali, the local name for *E. longifolia *(in which “Tongkat” meaning “walking stick” refers to the presence of long twisted roots), is known to be enriched with many valuable phytochemicals. More than 65 bioactive compounds have been isolated and characterized, including *β*-carboline and canthine-6-one alkaloids, quassinoids (laurycomalactone, eurycomalactone, longilactone, eurylactone, and eurycomanone), squalene derivatives, tirucallane triterpenes, mucopolysaccharides, biphenylneolignans, and eurypeptides [3–5]. Alkaloids and quassinoids comprise majority of the chemical composition and exhibit antimalarial [4], antioxidant [5], antiosteoporotic [6], antidiabetic [7], anticancer [8], antipyretic, and antianxiety activities, some of which have been demonstrated by pharmacological studies. As a health supplement ingredient, the efficacy of *E. longifolia *in restoring energy, improving muscle strength, alleviating aging males' symptoms [9], and acting as an aphrodisiac on late-onset hypogonadism management [10], has been highlighted. Water-soluble* E. longifolia *extract was reported to be able to enhance male fertility (with regards to higher semen volumes, spermatozoa count, and motility) in rodents [11, 12] and in human trials [13–15]. Scientific evidence proved these efficacies that resulted from the increase in testosterone levels via hypothalamic-pituitary-gonadal axis stimulation [12]. Eurycomanone, the major quassinoids in *E. longifolia*, enhances testosterone steroidogenesis at the leydig cells by inhibiting aromatase conversion of testosterone to oestrogen [16]. Besides, eurypeptides have been shown to activate the 17*α*-hydroxylase (CYP17 enzyme), triggering the biosynthesis of dehydroepiandrosterone which can act on androgen receptors to initiate the conversion of androstenedione to testosterone [10, 13, 17]. Therefore, *E. longifolia* supplementation may be an alternative for testosterone replacement therapy and the bone volume restoration from androgen deficient osteoporosis [18–21], or it may be able to improve certain mood state parameters in elders [22].

Although herbal medicines rely on remedies of natural origin and have fewer side effects than allopathic medicines, the use of herbal remedies is not always safe. Hence, concerns about their safety and toxicity are increasing, especially within the context of chronic or cumulative dosing [23]. Numerous commercial *E. longifolia* supplements do not provide their information about bioactive constituents, extraction methodology, and purity. In general, alcohol extracts (usually enriched eurycomalactone) are more toxic than water extracts [24, 25]. Although oral toxicity studies show that the lethal dose 50 (LD_50_) of *E. longifolia* water extract is at the dosage of 2 g/kg b.w. (acute), and that the no observed adverse effect level (NOAEL) is greater than 1 g/kg b.w. (28-day subacute feeding) [26] however, to our knowledge, these toxicological reports are incomplete and insufficient for demonstrating either long-term safety or prediction of acceptable daily intake (ADI). To establish evidence-based data, acute, subacute, and subchronic toxicity studies were conducted after single, 4-week, and 13-week repeated oral dosing in Wistar rats, and genotoxic parameters were also evaluated *in vitro* and *in vivo*. This study was performed in compliance with the testing guidelines of the Organization for Economic Cooperation and Development (OECD) and the Taiwan Food and Drug Administration.

## 2. Materials and Methods

### 2.1. *E. longifolia* Powdered Root Preparation

The powder of *E. longifolia* roots used in this study was supplied by Exclusive Mark (M) Sdn Bhd, Malaysia. Roots, at least 3 inches in diameter, were harvested, washed to remove stones and soil, and sliced by hand. The slices were dried in an oven at 110°C for 2 h and then crushed and ground to a fine powder. The powdered root was vibrated through a 150-grade mesh and submitted for quality control. The dry powder was stored at room temperature and resuspended in sterile water at the time of use.

### 2.2. *Salmonella*/Microsome Reversion Assay: Ames Test

The *Salmonella typhimurium* histidine auxotrophs TA98, TA100, TA1537, TA1535, and TA102 were purchased from Moltox (TM, USA). Mutagenicity was determined by incorporation method in the presence and absence of S9 metabolic activation [27]. One hundred microliters of each test bacterial culture (10^9^ cells/mL), 2 mL soft agar (0.6% agar, 0.5% NaCl, 5 mM histidine, and 50 mM biotin, pH 7.4, 40–50°C), 0.5 mL S9 mixture (if necessary), and test compounds were mixed well in a test tube. Immediately after mixing, the sample was poured into a minimal agar plate (1.5% agar, Vogel-Bonner E medium containing 2% glucose). Plates were incubated for 48 h at 37°C in the dark. The revertant colonies were counted macroscopically. A compound was considered positive for mutagenicity only when (i) the number of revertants was at least double the spontaneous yield, (ii) a statistical significance (*P* ≤ 0.05) was found, and (iii) a reproducible positive dose response was present.

### 2.3. Chromosomal Aberration

Chinese hamster ovary epithelial cells (CHO-K1, ATCC: CCL-61) were plated in 6 cm dishes at 5 × 10^5^ cells/plate for the 24 h treatment group. After overnight incubation, cells were treated with vehicle, mitomycin C (1 *μ*g/mL), benzo(a)pyrene (5 *μ*g/mL), or test compounds (1.25, 2.5, or 5 mg/mL) for 3–24 h, with or without S9. Then, 3 h after the end of the treatment time, 0.1 *μ*g/mL colcemid was administered, and metaphase chromosomes were prepared as previously described [27]. After trypsinization, cells were treated with 0.9% sodium citrate at 37°C for 10 min, fixed in Carnoy's solution (methanol : acetic acid, 3 : 1), and spread on glass slides. Specimens were stained with 3% Giemsa solution in 0.07 M phosphate buffer (pH 6.8) for 30 min. For determination of chromosome aberrations (e.g., chromosome-type gap (G), break (B), and dicentric (D); chromatid-type gap (g), break (b), and exchange (e)), a total of 100 well-spread diploid M1 metaphase cells per treatment were classified and scored. Data were expressed as the total mean number of chromosomal aberrations per treatment ± SEM from 300 cells scored in 3 independent experiments.

### 2.4. Animal Husbandry

Adult 4–6-week-old male and female Wistar rats, weighing 170–200 g, were obtained from BioLASCO Ltd. (Taipei, Taiwan). Male 6-week-old ICR mice were obtained from the Animal Center of National Taiwan University (Taipei, Taiwan). Animals were separated by sex, housed 3-4 per cage, quarantined, and acclimated for 1 week in the housing room under a 12 h light/dark cycle at 23 ± 1°C and 39–43% relative humidity. Water and food (Rodent LabDiet 5001; PMI Nutrition International, LLC, Richmond, IN, USA) were available ad libitum. All procedures involving the use of animals were in compliance with the “Guide for the Care and Use of Laboratory Animals” (National Academy of Sciences Press, 1996) and approved by the Institutional Animal Care and Use Committee at our institution (Approval no. LAC-97-0146).

### 2.5. Micronucleus Assay

Male ICR mice (*N* = 5) were administered test compounds orally by gavage at doses of 0.6, 1.2, and 2.0 g/kg body weight. Negative controls received the vehicle, and positive controls were administered an intraperitoneal injection of 200 mg/kg body weight cyclophosphamide. Peripheral blood (10–20 *μ*L) was sampled from the tail vein at 24, 48, and 72 h after-dosing, smeared on a glass microscope slide, and stained with acridine orange (40 *μ*g/mL). The prepared slides were immediately examined by fluorescence microscopy. The ratio (%) of polychromatic erythrocytes (PCEs) to total erythrocytes and the frequency of micronucleated polychromatic erythrocytes (MNPCEs; *‰*) were calculated by counting a total of 1000 erythrocytes or PCEs per animal, respectively.

### 2.6. Single-Dose Acute Toxicity Study

Forty healthy Wistar rats were divided into 5 groups (4 males and 4 females per group). The first group (control) received sterile water, given orally. Groups 2–5 were orally treated with test compounds in doses of 1, 2, 4, and 6 g/kg, respectively. Animals were observed for general behavioral and body weight changes, hazardous symptoms, and mortality for a 14-day period after treatment. At the end of the study (on day 14), rats were anesthetized with isoflurane, blood (for serum biochemistry analysis) was collected from the abdominal aorta, and gross necropsies were conducted. 

### 2.7. 4-Week Subacute and 13-Week Subchronic Toxicity Studies

Healthy Wistar rats were randomly divided into 4 groups. Group 1 (control) animals received vehicle treatment by oral gavage throughout the course of the study. Animals in groups 2–4 were orally administered test compound at doses of 0.6, 1.2, and 2 g/kg body weight/day for 4 weeks (10 males per group) or 13 weeks (10 males + 10 females per group). Body weight, food consumption, and water intake (by subtracting the weight of the remaining food/water from the weight of the supplied food/water) were recorded weekly. Mortality and signs of abnormalities were observed twice daily. At the end of the study, rats were fasted overnight (with water still supplied ad libitum) and anesthetized with isoflurane. Blood samples were obtained from the abdominal aorta using capillary tubes for hematological, clotting, and serum biochemical studies. 

### 2.8. Hematological, Clotting, and Serum Biochemical Analysis

Hematological profiles were obtained using an automatic analyzer (Sysmex K-4500, TOA Medical Electronics Co., Kobe, Japan). Before serum biochemistry (Express Plus automatic clinical chemistry analyzer, Siemens Medical Diagnostics) and clotting (AMAX 200, Trinity Biotec Plc., Ireland) analysis, serum and plasma samples were obtained by centrifugation at 1500 ×g for 10 min. Hematological and serum biochemical analyses were conducted according to the OECD Guidelines for the Testing of Chemicals no. 407 and no. 408. 

### 2.9. Urinalysis

Urinalyses were conducted during the last 4 days of the testing period. Changes in pH, protein and glucose content, specific gravity, uric acid composition, and the presence of occult blood or ketones were assessed (Miditron Junior, Roche, Diagnostics Gmbh, Mannheim, German). Samples were also examined microscopically for the presence of urinary sediments.

### 2.10. Gross Necropsy

Gross necropsies were performed to analyze macroscopic external and internal features (OECD Guidelines for the Testing of Chemicals no. 407 and no. 408). Vital organs were carefully removed, defatted, and individually weighed and then fixed in 10% neutral buffered formalin. Organ wet weights were expressed in absolute and relative terms (g and g/100 g body weight or brain weight, resp.).

### 2.11. Histopathology

The biopsy sections were prepared as described in Supplementary Materials available online at http://dx.doi.org/10.1155/2013/102987 histopathological report and examined by an experienced pathologist. Microscopic features of the organs of male and female rats were compared in the control and 2 g/kg body weight-treated groups.

### 2.12. Statistical Analysis

All values are expressed as the mean ± SD. Comparisons between groups were performed using one-way analysis of variance (ANOVA) followed by Dunnett multiple comparison tests using SPSS statistical software (DrMarketing Co. Ltd., New Taipei City, Taiwan). A *P* value of <0.05 was considered significant.

## 3. Results

### 3.1. Neither Mutagenicity nor Clastogenicity Was Caused by *E. longifolia* Powdered Root Treatment *In Vitro* and *In Vivo*

In the Ames test, on increase in the number of revertants was observed in the positive control group (*P* < 0.05). After treatment with various concentrations of *E. longifolia *powdered root, none of the testing concentrations (5, 2.5, 1.25, 0.625, or 0.3125 mg/plate) resulted in a clear growth in the number of colonies with or without S9 metabolic activation in all 5 test strains (TA97, TA98, TA100, TA102, and TA1535; Table 1). 

According to the cell viability assay, the selected testing concentrations (1.25, 2.5, and 5 mg/mL) were less than the LC_50_ (Supplementary Table 1). Compared to positive control treatments with mitomycin C (1 *μ*g/mL, without S9 activation) or benzo(a)pyrene (5 *μ*g/mL; with S9 activation), which showed abnormal increases in the number of damaged chromosomes (*P* < 0.05), no increases or irregularities in chromosome aberrations were recorded in all test treatment groups (Table 2). 

A reduction in the ratio of PCEs to total erythrocytes (%) and an increase in the frequency of MNPCEs (‰) were found in the positive control group (*P* < 0.05). The proportion of PCEs and the frequency of MNPCEs in treated (0.6, 1.2, and 2 g/kg b.w.) groups were not statistically different from those of the negative control animals (Table 3). These data indicated that neither mutagenicity nor clastogenicity was caused by *E. longifolia *powdered root treatments.

### 3.2. No Deaths or Abnormalities Were Observed in the Single-Dose Acute Toxicity Study

No deaths or hazardous signs were recorded in rats during the 14-day observation period for rats orally treated with *E. longifolia *powdered root (1, 2, 4, and 6 g/kg b.w.). Hematological and serum biochemical profiles of both sexes, as shown in Supplementary Table 2, remained unaffected.

### 3.3. *E. longifolia* Powdered Root Treatment Was Nontoxic but Induced Pharmacological Effects in Subacute and Subchronic Toxicity Studies

No treatment-related mortality or clinical signs of toxicity were observed throughout the 4- or 13-week consecutive testing period (at 0, 0.6, 1.2, and 2 g/kg b.w.). No statistically significant changes were found in body weight (Supplementary Table 3), food consumption, or water intake (data not shown), regardless of sex or treatment. Most hematology measures (WBC, RBC, Hb, HCT, PLT, MPV, RDW, and Fbg) in treated rats were not significantly different from controls, with the exception of marginal variations in certain parameters (MCHC and MCV; Table 4). The PT and APTT of treated males (in both the 4-week and 13-week treatment groups) were significantly decreased compared to control male rats (*P* < 0.05), whereas the PT and APTT were not different in control versus treated female rats (Table 4 and Supplementary Table 4). Serum biochemistry profiles are shown in Table 5. *E. longifolia *powdered root had no effects on serum electrolytes, such as calcium, potassium, sodium, and chloride, but did cause marginal variations in phosphate and magnesium. No statistically significant differences in liver function parameters (ALT, ALP, and GGT) were noted. However, the activities of AST, CPK, and LDH were reduced in a dose-dependent manner in both 4-week and 13-week treated males (*P* < 0.05). Dose-dependent reductions in kidney function parameters, creatinine, and BUN were noted in male rats treated for 13 weeks (the reduction in BUN was also noted in male rats in the 4-week treatment group) (*P* < 0.05). No similar changes in AST, CPK, LDH, BUN, or creatinine were found in the female groups. Serum cholesterol was significantly decreased in both male and female rats after 4 and 13 weeks of administration (*P* < 0.05). No significant changes in total protein, globulin, albumin, glucose, triglycerides, or amylase were noted.

Semiquantitative urinalysis (including analysis of urine volume, specific gravity, pH, urobilirubin, protein, glucose, urea acid, ketone, nitro salt, and occult blood) and urinary sediments did not reveal any relevant changes following 4- or 13-week administration of *E. longifolia *powdered root (Supplementary Table 6; data are not shown). 

### 3.4. Treatment with *E. longifolia* Powdered Root Caused no Macropathological or Histopathological Lesions in Subacute and Subchronic Toxicity Studies

Absolute and relative tissue weights (% ratio to body weight or brain weight) were not altered by 4- or 13-week treatments (Supplementary Table 5 and Table 6). Macroscopic examination of the vital organs of treated animals revealed no abnormalities in color or texture when compared with organs of the control group. Histological evaluation of organs did not reveal any pathological lesions in groups receiving the highest dosage (Supplementary information: histopathological report). 

## 4. Discussion

The safety of traditional herbs has been mostly determined by empirical experience. However, the therapeutic dose range of these herbs should be reanalyzed after specific processing procedures (e.g., extraction, dehydration, and capsule or tincture formulations). In the current study, we first determined that the LD_50_ of *E. longifolia* powdered root, as measured by oral single-dose administration (the acute toxicity study), was more than 6 g/kg b.w. in rats. *E. longifolia* powdered root (6 g/kg b.w.) did not cause any acute toxicities (defined as a rapid onset of symptoms within hours or days) or abnormalities in serum biochemical, hematological, and clotting profiles. However, in previous studies, the reported LD_50_ of *E. longifolia *in mice was 1.89 g/kg (orally administered, 50% aqueous ethanolic extract), and on further fractionation, LD_50_ values were 1.36 g/kg (n-butanol fraction), 2.31 g/kg (diethyl ether fraction), and 5.27 g/kg (water fraction). Eurycomanone, the main component identified in the n-butanol fraction, is the most toxic constituent of *E. longifolia* (LD_50_ = 0.05 g/kg) [28]. These discrepancies in the LD_50_ values reported in different studies resulted from variations in the testing system, especially with respect to animal species and preparation of test compounds. Studies have shown that extracts prepared using organic solvents were more toxic than water extracts [24, 25, 28, 29]. 

In cumulative, subacute (4-week), and subchronic (13-week) studies,* E. longifolia* powdered root was found to be nontoxic to liver/renal function, and hematological profiles, lipid profiles, body electrolytes, and coagulation factors were not altered. However, dose-related (as well as dosing period-related) changes in several serum biochemical parameters were observed. Decreases in LDH, CPK, AST, CRE, and BUN levels in treated groups (*P* < 0.05) explain the cytoprotective potential of *E. longifolia*, especially in the liver and kidneys. This cytoprotective potential may be due to the pharmacological effects of superoxide dismutase, *β*-carboline alkaloids, and squalene derivatives. These compounds are known to be effective scavengers of reactive oxygen species and have significant protective effects against oxidative damage, lipid peroxidation, and inflammation [5, 30–33]. The powdered root of *E. longifolia *also caused decreases in PT, APTT (in plasma), and cholesterol levels (in serum) in this study (*P* < 0.05) and improved HDL levels in volunteers in a separate study [34]. *β*-Carboline alkaloids can act as antithrombotic agents that inhibit platelet coagulation [35]. Squalene is a well-known precursor of cholesterol biosynthesis and has therapeutic potential in the prevention of hypercholesterolemia and cardiovascular disease [33]. More interestingly, except in the case of serum cholesterol level, these changes were observed only in dosed males. In previous studies on the aphrodisiac properties of *E. longifolia*, an enhancement in sexual behavior was found only in male rats and mice [11, 36], and an increase in testosterone production was found in men [13]. No similar report in women (or female rodents) has been documented. *E. longifolia* supplementation failed to preserve the bone microarchitecture in orchidectomized rat model [20]. These data suggest that the pharmacological effects of *E. longifolia* may be sex specific.

In a double-blind, placebo-controlled clinical trial (using 200–600 mg/kg water extracted *E. longifolia* root) in volunteers with type 2 diabetes, this herbal treatment had obvious effects on the lowering of blood glucose [34]. Similar results were also observed in hyperglycemic rats treated with aqueous *E. longifolia* extracts (50–150 mg/kg) or freeze-dried powder (6.25 mg/kg); no reduction in blood glucose was noted in normoglycemic rats with the same treatment, suggesting that *E. longifolia* may have significant antidiabetic therapeutic potential [7]. In this study, blood glucose levels were unaffected after 4 or 13 weeks of *E. longifolia* powdered root treatment.

Aristolochic acid nephropathy is a typical example of chronic toxicity due to cumulative genetic damage. Symptoms of this damage can be delayed for years, even after stopping the use of the toxic herbs [23]. *β*-Carboline alkaloids have been reported to be mutagenic and genotoxic [37, 38]. In a previous study, although the revertants induced by *E. longifolia* (250 *μ*g/mL; methanol/chloroform extracts) were not significantly different from the control, a higher level of mutagenicity was noted, suggesting that some active components of *E. longifolia* may be mutagenic but are present at very low concentrations [39]. In the current study, mutagenicity (Ames test) and clastogenicity (chromosomal aberrations *in vitro *and micronucleus test *in vivo*) were determined in accordance with international guidelines. Neither mutagenic nor clastogenic effects were found *in vitro* (at concentrations up to 5 mg/plate or 5 mg/mL) or *in vivo* (at doses up to 2 g/kg b.w.), suggesting that *E. longifolia* powdered root is not genotoxic.

In addition, *E. longifolia* crude extracts displayed cytotoxic activity (with IC_50_ values ranging from 10 to 15 *μ*g/mL in different cell types). Isolated quassinoids, such as eurycomalactone and eurycomanone, were found to be highly cytotoxic against a panel of cancer cell lines (with IC_50_ values ranging from 0.93 to 1.1 *μ*M; IC_50_ = 3.8 *μ*g/mL in HepG2 cells, IC_50_ = 15.2 *μ*g/mL in MCF-7 cells), but they had a certain degree of cytoselectivity in noncancerous or normal cells (IC_50_ = 17 *μ*g/mL in Chang's liver cells, IC_50_ = 66.3 *μ*g/mL in MCF-10A cells) [40, 41]. The involvement of p53/Bax signaling and downregulation of cancer markers (such as Bcl-2 heterogeneous nuclear ribonucleoprotein A2/B1, prohibitin, and annexin) has been shown to be involved in eurycomanone-induced apoptosis [40, 42] and anchorage-independent growth inhibition [8]. Although *E. longifolia* extracts have been shown to have strong cytotoxic potential, no cytotoxic biomarkers were noted after 13-week exposure in our current study. In past studies, high *in vivo* cytotoxicity was identified in rodents injected intraperitoneally with *E. longifolia* (2.44 *μ*mol/kg/day for 3 consecutive days). Highly toxic quassinoids showed poor oral bioavailability in rat models [43] and bypassed first-pass liver metabolism when given by intraperitoneal. Hence, toxicity decreased by approximately 100-fold if taken orally [3]. This finding supports that, for efficacy and safety purposes, *E. longifolia* powdered root should only be consumed orally.

Currently, long-term consumption of *E. longifolia* root extract has been associated with sleep apnea, facial flushing, pressure in the testicles, and hyperaggressiveness. However, no scientific evidence supporting these side effects has been found. Furthermore, no detailed clinical evidence supports the use of specific doses of *E. longifolia*. According to the recommended intake for humans, a dosage of 100–600 mg/day (water extract) exerted pharmacological effects in men after 2–8 weeks of supplementation [34], and a reference daily dose level of 270–350 mg/kg was found to be rather safe [3]. In this study, toxicity assessments were based on standard guidelines that can be easily comparable between laboratories. Our data showed that neither acute nor subchronic/delayed toxicity was found. Therefore, a dose of 2 g/kg b.w./day of *E. longifolia* powdered root, generated from our 13-week subchronic toxicity study, can be considered as a no observed adverse effect level (NOAEL). The calculated acceptable daily intake (ADI) is up to 1.2 g/60 kg adult/day (with a safety factor of 100), equivalent to 12 times the recommended intake dosage for humans (100 mg/60 kg adult/day). This information will be useful in the development of *E. longifolia* products and management of the safety profile for this herbal medicine.

## 5. Conclusion

*Eurycoma longifolia* powdered root is not genotoxic. The acute oral LD_50_ was more than 6 g/kg b.w. No test compound related toxicity was found after 13-week consecutive exposure. Anticoagulation and liver/kidney cytoprotection were noted in treated males. The calculated acceptable daily intake was up to 1.2 g/adult/day.

## Supplementary Material

The cytotoxicity evaluation (MTT assay) on *E. longifolia* powdered root treated CHO-K1 cells.
The record of 14-day acute toxicity study of *E. longifolia* powdered root .
The record of mean body weight in 13-week subchronic toxicity study (*E. longifolia* powdered root).
Hematology and serum biochemistry profiles of subacute toxicity after 4-week *E. longifolia* powdered root treatment.
The absolute and relative tissue weights of subchronic toxicity after 4-week *E. longifolia*powdered root treatment.
Urinlysis of subchronic toxicity after 13-week *E. longifolia* powdered root treatment.

## Figures and Tables

**Table 1:** Mutagenicity assay of *E. longifolia* powdered root* in vitro* (Ames test).

	TA98	TA100	TA102	TA1535	TA1537
	Without S9 metabolic activation				
Negative^1^	22.0 ± 3.0	141.7 ± 34.2	202.7 ± 15.7	11.3 ± 0.6	6.0 ± 1.0
Positive^2^	1026.7 ± 254.0*	3976.0 ± 644.1*	1274.7 ± 185.4*	1234.3 ± 53.8*	762.3 ± 655.3*
*E. longifolia* powdered root (mg/plate)					
0.3125	18.0 ± 0.0	204.3 ± 26.0	109.0 ± 6.9	14.3 ± 1.2	8.0 ± 1.7
0.625	21.7 ± 5.8	187.3 ± 48.8	98.0 ± 10.5	15.7 ± 3.1	5.7 ± 0.6
1.25	20.0 ± 0.0	187.0 ± 24.3	103.7 ± 24.7	18.3 ± 5.8	6.3 ± 2.1
2.5	13.3 ± 3.2	195.3 ± 37.5	110.3 ± 12.4	16.3 ± 7.6	8.3 ± 3.1
5	18.7 ± 2.5	184.0 ± 14.8	98.0 ± 12.8	22.0 ± 1.0	8.7 ± 0.6

	With S9 metabolic activation				
Negative^1^	29.0 ± 3.6	83.0 ± 4.4	160.7 ± 18.3	14.3 ± 0.6	9.0 ± 1.0
Positive^2^	100.3 ± 12.0*	599.7 ± 49.4*	1565.7 ± 59.8*	960.3 ± 71.8*	857.7 ± 20.4*
*E. longifolia* powdered root (mg/plate)					
0.3125	23.0 ± 5.3	159.7 ± 14.0	181.7 ± 8.5	12.3 ± 6.4	11.7 ± 3.5
0.625	23.0 ± 2.7	171.7 ± 18.2	129.7 ± 31.3	10.3 ± 4.7	7.5 ± 2.1
1.25	21.7 ± 2.1	201.3 ± 9.3	157.7 ± 12.5	13.0 ± 2.7	10.7 ± 1.2
2.5	20.3 ± 3.2	161.3 ± 35.4	167.3 ± 15.1	9.3 ± 6.7	6.3 ± 3.8
5	20.7 ± 3.1	128.3 ± 29.5	163.3 ± 7.7	15.7 ± 6.4	7.3 ± 2.5

The values were presented as Mean ± S.D. (*N* ≥ 3). No significant changes of revertants were observed at any concentrations of test compound treatments in five tester strains. Statistical analysis: *P < 0.05 indicates a statistical difference with the control group by one-way ANOVA when the number of revertants was twice than control at TA98, TA100, and TA102 and triple than control at TA1537 and TA1535.

^1^Solvent (sterile water) was used as negative control.

^2^Positive control in w/o S9 plate: TA98 (4-nitro-o-phenylenediamine 2.5 *μ*g/plate); TA100 and TA1535 (sodium azide 5 *μ*g/plate); TA102 (mitomycin C 0.5 *μ*g/plate); TA1537 (9-aminoacridine 50 *μ*g/plate); in w/w S9 plate: TA100/TA102/TA1535/TA1537 (2-aminoanthrene 5 *μ*g/plate); TA98 (benzo(a)pyrene 5 *μ*g/plate).

**Table 2:** Chromosome aberration assay of *E. longifolia* powdered root *in vitro*.

	Aberrant cells (%)	Number of aberrations/100 cells^3-4^					
		G	B	D	g	b	e
	3 h treatment/without S9 metabolic activation						
Negative^1^	1.7 ± 0.6	0.3 ± 0.3	0.3 ± 0.3	0	0.7 ± 0.7	0.3 ± 0.3	0
Positive^2^	7.0 ± 2.7*	0.3 ± 0.3	0.3 ± 0.3	0.3 ± 0.3	1.7 ± 0.7	0.7 ± 0.3	3.7 ± 1.7
*E. longifolia* powdered root (mg/mL)							
1.25	1.0 ± 1.0	0	0	0	0.7 ± 0.3	0.3 ± 0.3	0
2.5	0.7 ± 0.6	0	0	0	0.3 ± 0.3	0.3 ± 0.3	0
5	1.3 ± 0.6	0	0	0	1.0 ± 0.6	0.3 ± 0.3	0

	3 h treatment/with S9 metabolic activation						
Negative^1^	0.7 ± 1.2	0	0.3 ± 0.3	0.3 ± 0.3	0	0	0
Positive^2^	5.7 ± 1.5*	0	2.0 ± 0.6	0.7 ± 0.7	1.7 ± 0.3	0.7 ± 0.7	0.7 ± 0.3
*E. longifolia* powdered root (mg/mL)							
1.25	1.3 ± 1.2	0	0	0	1.0 ± 0.6	0.3 ± 0.3	0
2.5	0.7 ± 0.6	0	0	0	0.3 ± 0.3	0	0.3 ± 0.3
5	0.7 ± 0.6	0.3 ± 0.3	0	0	0	0.3 ± 0.3	0

	24 h treatment/without S9 metabolic activation						
Negative^1^	0.7 ± 0.6	0	0	0	0	0.7 ± 0.3	0
Positive^2^	9.0 ± 1.0*	1.3 ± 0.3	0.7 ± 0.3	0	2.7 ± 0.9	2.3 ± 0.7	2.3 ± 0.7
*E. longifolia* powdered root (mg/mL)							
1.25	1.7 ± 0.6	0	0	0	0.3 ± 0.3	1.3 ± 0.3	0
2.5	1.3 ± 0.6	0	0	0	1.0 ± 0.7	0.3 ± 0.3	0
5	1.3 ± 0.6	0	0	0	0.7 ± 0.3	0.7 ± 0.3	0

^1^Solvent (sterile water) was used as negative control.

^2^Positive control in w/o S9 condition: mitomycin C 1 *μ*g/mL; in w/w S9 condition: benzo(a)pyrene 5 *μ*g/mL.

^3^The chromosomal aberrations were counted by 100 independent cells. For each treatment, at least 300 cells were examined as described in Section 2. The data are expressed as the Mean ± SEM (*N* = 3). Significant difference between control treated group at *P < 0.05 versus control by one-way ANOVA.

^4^G: chromosome gap; B: chromosome break; D: dicentric; g: gap; b: chromatid break; e: exchange.

**Table 3:** Micronucleus assay of *E. longifolia* powdered root *in vivo*.

Treatment	Dose	Frequency of micronucleated polychromatic erythrocyte (‰)			Ratio of polychromatic erythrocyte (PCE) to total erythrocytes (%)		
		Day 1	Day 2	Day 3	Day 1	Day 2	Day 3
Negative^1^	0	1.67 ± 0.58	1.33 ± 0.63	1.60 ± 0.76	4.00 ± 1.00	4.20 ± 0.84	4.40 ± 0.89
Positive cyclophosphamide (mg/kg b.w.)	200	10.53 ± 2.60***	12.27 ± 3.68***	10.33 ± 1.62***	1.20 ± 0.45***	1.00 ± 1.00***	0.80 ± 0.45***
*E. longifolia* powdered root (g/kg b.w.)	0.6	1.67 ± 0.34	1.13 ± 0.56	1.53 ± 0.65	5.60 ± 1.14	5.20 ± 0.84	5.40 ± 0.55
	1.2	1.80 ± 0.45	1.46 ± 0.50	1.13 ± 0.64	5.20 ± 0.84	5.40 ± 0.55	5.20 ± 0.84
	2.0	1.60 ± 0.37	1.93 ± 0.72	1.47 ± 0.38	5.20 ± 0.84	5.00 ± 0.71	5.20 ± 0.84

Both the ratios of polychromatic erythrocyte (PCE) to total erythrocytes (%) and the frequency of micronucleated polychromatic erythrocyte (%) in treated group were not statistically different from those of the negative control animals, suggesting that the treatment with *E. longifolia* powdered root did not cause erythropoietic cell toxicity and genotoxicity *in vivo*. Data were expressed as Mean ± S.D. (*N* = 5). Statistical analysis: *P < 0.05,  **P < 0.01, and ***P < 0.001 indicate a statistical difference with the control group by one-way ANOVA.

^1^Mice in negative control group received single gavage of vehicle (sterile water).

**Table 4:** Hematology analysis of subchronic toxicity after 13-week *E. longifolia* powdered root treatment.

Item (abbreviation)	Unit	Treatment (g/kg b.w.)							
		Male				Female			
		0	0.6	1.2	2	0	0.6	1.2	2
Red blood cell count (RBC)	10^6^/*μ*L	8.20 ± 0.38	8.26 ± 0.41	8.44 ± 0.38	8.41 ± 0.30	7.98 ± 0.17	7.91 ± 0.42	7.77 ± 0.37	8.37 ± 0.35
Hematocrit (HCT)	%	43.88 ± 1.27	43.78 ± 1.88	43.88 ± 2.23	43.01 ± 1.34	44.63 ± 1.42	43.94 ± 2.54	43.37 ± 3.06	44.78 ± 2.08
Hemoglobin (Hb)	g/dL	14.29 ± 0.46	14.33 ± 0.64	14.63 ± 0.79	14.52 ± 0.39	14.49 ± 0.47	14.40 ± 0.86	14.40 ± 0.85	15.02 ± 0.67
Mean corpuscular hemoglobin (MCH)	pg	17.43 ± 0.53	17.37 ± 0.41	17.33 ± 0.43	17.29 ± 0.55	18.16 ± 0.59	18.19 ± 0.54	18.52 ± 0.51	17.94 ± 0.32
MCH concentration (MCHC)	g/dL	32.57 ± 0.50	32.74 ± 0.65	33.36 ± 0.61*	33.76 ± 0.39*	32.47 ± 0.68	32.78 ± 0.77	33.24 ± 1.08	33.56 ± 0.74
Mean corpuscular volume (MCV)	fL	53.56 ± 1.62	53.06 ± 1.64	52.02 ± 1.26	51.20 ± 2.04*	55.95 ± 2.15	55.55 ± 1.62	55.79 ± 2.88	53.50 ± 1.21
Red cell distribution width (RDW)	fL	25.81 ± 2.05	25.01 ± 1.47	24.37 ± 1.32	24.26 ± 1.09	26.48 ± 1.83	25.89 ± 1.77	26.23 ± 1.92	24.52 ± 1.66
Mean platelet volume (MPV)	fL	7.70 ± 0.21	7.76 ± 0.23	7.48 ± 0.21	7.49 ± 0.24	8.37 ± 0.45	8.16 ± 0.51	8.23 ± 0.43	8.01 ± 0.46
Platelet distribution width (PDW)	fL	8.26 ± 0.36	8.32 ± 0.34	7.87 ± 0.47	7.84 ± 0.37	9.50 ± 0.73	9.15 ± 0.95	9.29 ± 0.66	9.06 ± 0.87
White blood cell count (WBC)	10^3^/*μ*L	2.62 ± 0.94	3.46 ± 1.18	4.44 ± 3.27	3.09 ± 1.59	2.03 ± 0.95	1.72 ± 1.20	1.61 ± 0.99	3.37 ± 1.40
Lymphocytes	10^3^/mm^3^	67.41 ± 10.10	67.71 ± 6.04	70.17 ± 15.34	69.97 ± 7.34	70.12 ± 12.07	66.23 ± 12.91	73.24 ± 5.40	76.94 ± 8.13
Neutrophils	10^3^/mm^3^	26.32 ± 9.82	8.32 ± 0.34	7.87 ± 0.47	7.84 ± 0.37	22.07 ± 7.87	27.98 ± 11.38	21.31 ± 5.20	17.83 ± 6.74
Monocytes	10^3^/mm^3^	3.24 ± 2.69	4.31 ± 2.40	3.03 ± 2.45	2.86 ± 2.05	4.76 ± 6.63	2.18 ± 1.96	3.25 ± 2.43	2.95 ± 1.35
Eosinophil	10^3^/mm^3^	3.03 ± 1.21	2.50 ± 0.46	2.42 ± 1.29	2.29 ± 0.76	3.05 ± 1.14	3.61 ± 2.02	2.20 ± 1.84	2.28 ± 1.11
Basophils	10^3^/mm^3^	0.00 ± 0.00	0.00 ± 0.00	0.02 ± 0.06	0.00 ± 0.00	0.00 ± 0.00	0.00 ± 0.00	0.00 ± 0.00	0.00 ± 0.00
Platelets count (PLT)	10^3^/*μ*L	635.9 ± 67.6	678.2 ± 66.3	732.6 ± 97.9	729.0 ± 89.1	752.3 ± 65.5	684.4 ± 115.9	845.7 ± 110.3	773.4 ± 108.6
Prothrombin time (PT)	Sec	12.33 ± 1.23	12.51 ± 1.05	11.11 ± 0.84*	10.59 ± 0.19*	10.34 ± 0.72	10.36 ± 0.42	10.87 ± 0.99	10.68 ± 0.47
Partial thromboplastin time (APTT)	Sec	32.24 ± 5.00	32.45 ± 6.62	27.94 ± 3.07*	23.43 ± 2.32*	29.51 ± 4.84	30.39 ± 5.12	31.08 ± 3.65	31.16 ± 3.69
Fibrinogen (Fbg)	mg/dL	183.4 ± 27.6	185.0 ± 36.4	176.7 ± 35.5	168.6 ± 36.9	126.3 ± 13.6	125.1 ± 18.6	125.6 ± 10.6	128.3 ± 16.4

Hematology profiles were within normal range (Charles River, 2008) and unaffected by the *E. longifolia* powdered root ingestions. Data were expressed as Mean ± S.D. (*N* = 8–10). Statistical analysis: *P < 0.05 indicates a statistical difference with the control group by one-way ANOVA.

**Table 5:** Serum biochemistry analysis of subchronic toxicity after 13-week *E. longifolia* powdered root treatment.

Item (abbreviation)	Unit	Treatment (g/kg b.w.)							
		Male				Female			
		0	0.6	1.2	2	0	0.6	1.2	2
Calcium	mg/dL	10.39 ± 0.46	10.37 ± 1.17	10.02 ± 1.13	9.78 ± 0.63	9.25 ± 0.59	9.29 ± 0.97	10.15 ± 0.48	9.87 ± 0.90
Chloride	mmol/L	105.10 ± 7.29	104.90 ± 13.49	107.00 ± 10.24	101.50 ± 6.17	101.00 ± 6.48	99.20 ± 10.50	100.70 ± 6.95	100.00 ± 8.37
Potassium	mmol/L	5.67 ± 0.74	5.49 ± 1.20	5.63 ± 0.62	5.05 ± 0.65	4.49 ± 0.46	4.53 ± 0.83	4.31 ± 0.75	4.35 ± 0.62
Sodium	mmol/L	146.90 ± 9.12	146.90 ± 17.11	150.50 ± 13.53	143.40 ± 8.15	139.00 ± 9.46	132.00 ± 19.68	140.90 ± 9.10	137.50 ± 12.08
Magnesium	mEg/L	1.68 ± 0.25	1.90 ± 0.25	1.95 ± 0.37	1.90 ± 0.25	1.46 ± 0.20	1.51 ± 0.35	1.88 ± 0.28*	1.86 ± 0.28*
Glucose	mg/dL	135.10 ± 17.46	143.90 ± 26.80	146.40 ± 14.06	126.70 ± 20.44	142.30 ± 23.36	113.30 ± 24.69	128.70 ± 21.23	131.70 ± 20.78
Alanine aminotransferase (ALT)	U/L	22.40 ± 4.01	21.90 ± 3.28	22.10 ± 4.61	21.80 ± 4.18	26.50 ± 14.20	21.10 ± 4.53	21.80 ± 3.88	22.70 ± 7.99
Aspartate aminotransferase (AST)	U/L	99.67 ± 13.31	94.60 ± 21.74	76.50 ± 16.66*	73.60 ± 12.69*	103.44 ± 18.93	99.56 ± 17.26	92.60 ± 18.02	86.56 ± 18.24
Alkaline phosphatase (ALP)	U/L	35.20 ± 4.02	35.40 ± 8.55	36.60 ± 7.93	34.40 ± 5.80	14.60 ± 2.55	16.40 ± 4.65	21.00 ± 7.47	19.60 ± 4.60
Gamma glutamyl transpeptidase (GGT)	mg/dL	0.62 ± 0.38	0.66 ± 0.61	0.81 ± 0.40	0.80 ± 0.51	0.35 ± 0.37	0.53 ± 0.44	0.30 ± 0.48	0.35 ± 0.38
Creatinine	mg/dL	0.76 ± 0.07	0.74 ± 0.10	0.67 ± 0.08*	0.61 ± 0.03*	0.68 ± 0.08	0.66 ± 0.08	0.64 ± 0.10	0.65 ± 0.11
Blood urea nitrogen (BUN)	mg/dL	23.40 ± 1.26	23.78 ± 4.41	20.33 ± 3.61*	16.80 ± 3.16*	22.60 ± 4.33	23.40 ± 3.89	25.50 ± 4.93	19.60 ± 4.84
Albumin	g/dL	3.15 ± 0.21	3.20 ± 0.35	3.28 ± 0.31	3.25 ± 0.20	3.51 ± 0.38	3.38 ± 0.53	3.65 ± 0.36	3.45 ± 0.47
Globulin	U/L	2.66 ± 0.13	2.59 ± 0.33	2.67 ± 0.40	2.53 ± 0.28	2.13 ± 0.20	2.13 ± 0.25	2.39 ± 0.14	2.24 ± 0.24
Total protein	g/dL	5.81 ± 0.31	5.79 ± 0.62	5.95 ± 0.66	5.78 ± 0.42	5.64 ± 0.54	5.51 ± 0.76	6.04 ± 0.42	5.69 ± 0.65
Cholesterol	mg/dL	92.97 ± 12.07	88.39 ± 23.38	70.86 ± 12.62*	60.83 ± 8.53*	81.45 ± 15.40	72.00 ± 17.55	70.50 ± 19.91	56.77 ± 16.35*
Triglycerides	mg/dL	44.50 ± 12.70	43.10 ± 12.72	42.90 ± 11.52	42.40 ± 8.15	44.50 ± 10.63	40.20 ± 9.55	39.00 ± 12.65	41.60 ± 6.82
Creatine phosphate kinase (CPK)	U/L	293.2 ± 104.0	215.9 ± 104.7	158.1 ± 119.0*	124.3 ± 70.2*	356.5 ± 112.5	339.7 ± 85.9	258.4 ± 77.1	280.9 ± 116.7
Lactate dehydrogenase (LDH)	U/L	652.7 ± 170.9	444.0 ± 227.5*	304.7 ± 93.2*	229.9 ± 93.2*	628.3 ± 155.5	691.6 ± 133.5	539.0 ± 128.9	575.9 ± 282.2
Amylase	U/L	704.1 ± 134.7	697.5 ± 103.0	665.7 ± 259.4	682.2 ± 81.7	513.2 ± 58.3	467.5 ± 90.1	486.9 ± 93.1	475.9 ± 95.3

Most serum biochemical parameters were within normal range (Charles River, 2008), except the pharmacological effects in AST (SGOT), creatinine, BUN, CPK, and LDH in treated males; cholesterol in both treated genders, after 13-week *E. longifolia* powdered root ingestions. Data were expressed as Mean ± S.D. (*N* = 8–10). Statistical analysis: *P < 0.05, **P < 0.01, and ***P < 0.001 indicate a statistical difference with the control group by one-way ANOVA.

**Table 6:** The absolute and relative tissue weights of subchronic toxicity after 13-week *E. longifolia* powdered root treatment.

		Treatment (g/kg b.w.)							
		Male				Female			
		0	0.6	1.2	2	0	0.6	1.2	2
Brain									
Absolute weight	Gram (10^−3^)	2.16 ± 0.11	2.18 ± 0.10	2.17 ± 0.10	2.10 ± 0.16	2.06 ± 0.06	2.03 ± 0.11	2.04 ± 0.06	2.07 ± 0.07
Ratio per body weight		4.83 ± 0.50	4.87 ± 0.37	4.95 ± 0.53	4.78 ± 0.55	7.63 ± 0.43	7.54 ± 0.68	7.76 ± 0.90	7.79 ± 0.60
Adrenals									
Absolute weight	Gram (10^−3^)	0.02 ± 0.00	0.03 ± 0.00	0.02 ± 0.01	0.03 ± 0.00	0.03 ± 0.01	0.03 ± 0.00	0.04 ± 0.01	0.03 ± 0.01
Ratio per body weight		0.05 ± 0.01	0.06 ± 0.01	0.05 ± 0.01	0.06 ± 0.01	0.13 ± 0.02	0.12 ± 0.02	0.14 ± 0.04	0.12 ± 0.04
Ratio per brain		0.01 ± 0.00	0.01 ± 0.00	0.01 ± 0.00	0.01 ± 0.00	0.02 ± 0.00	0.02 ± 0.00	0.02 ± 0.00	0.02 ± 0.0
Heart									
Absolute weight	Gram (10^−3^)	1.19 ± 0.17	1.19 ± 0.10	1.19 ± 0.13	1.27 ± 0.15	0.82 ± 0.07	0.83 ± 0.06	0.82 ± 0.11	0.82 ± 0.07
Ratio per body weight		2.63 ± 0.33	2.66 ± 0.21	2.70 ± 0.25	2.87 ± 0.27	3.06 ± 0.29	3.07 ± 0.22	3.12 ± 0.40	3.05 ± 0.19
Ratio per brain		0.55 ± 0.08	0.55 ± 0.05	0.55 ± 0.06	0.60 ± 0.06	0.40 ± 0.04	0.41 ± 0.03	0.41 ± 0.06	0.40 ± 0.04
Kidneys									
Absolute weight	Gram (10^−3^)	1.18 ± 0.08	1.22 ± 0.08	1.19 ± 0.18	1.19 ± 0.08	0.83 ± 0.05	0.86 ± 0.11	0.86 ± 0.14	0.76 ± 0.07
Ratio per body weight		2.63 ± 0.23	2.73 ± 0.20	2.69 ± 0.16	2.72 ± 0.27	3.08 ± 0.22	3.20 ± 0.47	3.22 ± 0.39	2.86 ± 0.17
Ratio per brain		0.55 ± 0.04	0.56 ± 0.04	0.55 ± 0.08	0.57 ± 0.06	0.40 ± 0.02	0.42 ± 0.04	0.42 ± 0.07	0.37 ± 0.04
Liver									
Absolute weight	Gram (10^−3^)	10.82 ± 1.23	10.84 ± 1.19	9.91 ± 1.37	10.02 ± 0.96	7.70 ± 1.00	7.89 ± 1.30	7.04 ± 1.18	7.05 ± 0.74
Ratio per body weight		24.03 ± 2.26	24.10 ± 1.62	22.37 ± 1.28	22.77 ± 2.40	28.48 ± 2.72	29.28 ± 5.06	26.47 ± 3.01	26.44 ± 2.18
Ratio per brain		5.02 ± 0.62	4.98 ± 0.59	4.57 ± 0.62	4.80 ± 0.57	3.75 ± 0.46	3.89 ± 0.66	3.46 ± 0.60	3.42 ± 0.40
Spleen									
Absolute weight	Gram (10^−3^)	0.80 ± 0.14	0.75 ± 0.11	0.78 ± 0.15	0.79 ± 0.10	0.50 ± 0.09	0.52 ± 0.08	0.56 ± 0.10	0.55 ± 0.04
Ratio per body weight		1.77 ± 0.28	1.67 ± 0.21	1.77 ± 0.34	1.81 ± 0.32	1.87 ± 0.35	1.93 ± 0.25	2.09 ± 0.30	2.07 ± 0.23
Ratio per brain		0.37 ± 0.07	0.34 ± 0.05	0.36 ± 0.06	0.38 ± 0.06	0.24 ± .04	0.26 ± 0.04	0.27 ± 0.05	0.27 ± 0.02
Testes									
Absolute weight	Gram (10^−3^)	1.60 ± 0.23	1.64 ± 0.13	1.52 ± 0.38	1.71 ± 0.13	0.04 ± 0.02	0.04 ± 0.01	0.04 ± 0.01	0.05 ± 0.02
Ratio per body weight		3.57 ± 0.66	3.65 ± 0.34	3.44 ± 0.82	3.92 ± 0.58	0.14 ± 0.07	0.14 ± 0.06	0.16 ± 0.05	0.19 ± 0.06
Ratio per brain		0.74 ± 0.14	0.75 ± 0.08	0.70 ± 0.16	0.82 ± 0.06	0.02 ± 0.01	0.02 ± 0.01	0.02 ± 0.00	0.02 ± 0.01
Thymus									
Absolute weight	Gram (10^−3^)	0.28 ± 0.04	0.29 ± 0.05	0.26 ± 0.06	0.23 ± 0.06	0.21 ± 0.03	0.20 ± 0.06	0.21 ± 0.08	0.19 ± 0.04
Ratio per body weight		0.63 ± 0.11	0.64 ± 0.10	0.58 ± 0.13	0.51 ± 0.10	0.77 ± 0.09	0.71 ± 0.19	0.78 ± 0.28	0.72 ± 0.13
Ratio per brain		0.13 ± 0.02	0.13 ± 0.02	0.12 ± 0.03	0.11 ± 0.03	0.10 ± 0.01	0.10 ± 0.03	0.10 ± 0.04	0.09 ± 0.02
Lung									
Absolute weight	Gram (10^−3^)	1.67 ± 0.25	1.68 ± 0.18	1.88 ± 0.35	1.68 ± 0.13	1.31 ± 0.17	1.39 ± 0.30	1.28 ± 0.12	1.30 ± 0.11
Ratio per body weight		3.71 ± 0.54	3.74 ± 0.35	4.29 ± 0.90	3.82 ± 0.32	4.84 ± 0.44	5.19 ± 1.37	4.86 ± 0.58	4.88 ± 0.35
Ratio per brain		0.77 ± 0.12	0.77 ± 0.10	0.87 ± 0.16	0.81 ± 0.09	0.64 ± 0.08	0.69 ± 0.16	0.63 ± 0.06	0.63 ± 0.06

After the 13-week treatment, the wet organ weights and related organ weight to body weight (or brain weight) remained unaffected as compared to control group. Data were expressed as Mean ± S.D. (*N* = 10). Statistical analysis: *P < 0.05,  **P < 0.01, and ***P < 0.001 indicate a statistical difference with the control group by one-way ANOVA.

## Abbreviations

PT: Prothrombin time
APTT: Partial thromboplastin time
BUN: Blood urea nitrogen
AST: Aspartate aminotransferase
SGOT: Serum glutamic-oxaloacetic transaminase
ALT: Alanine aminotransferase
SGPT: Serum glutamic-pyruvic transaminase
ALP: Alkaline phosphatase
GGT: Gamma glutamyl transpeptidase
CPK: Creatine phosphate kinase
LDH: Lactate dehydrogenase
NOAEL: No observed adverse effect level
ADI: Acceptable daily intake
LD_50_: Lethal dose 50
PCEs: Polychromatic erythrocytes
MNPCEs: Micronucleated polychromatic erythrocytes.
