# Supplementary material for: Evaluation of Acute 13-Week Subchronic Toxicity and Genotoxicity of the Powdered Root of Tongkat Ali (Eurycoma longifolia Jack)
Source: Evid Based Complement Alternat Med. 2013 Aug 25;2013:102987. doi: 10.1155/2013/102987 (PMC3767077; doi:10.1155/2013/102987)
Supplement: Supplementary file 1 — The cytotoxicity evaluation (MTT assay) on E. longifolia powdered root treated CHO-K1 cells. The record of 14-day acute toxicity study of E. longifolia powdered root . The record of mean body weight in 13-week subchronic toxicity study (E. longifolia powdered root). Hematology and serum biochemistry profiles of subacute toxicity after 4-week E. longifolia powdered root treatment. The absolute and relative tissue weights of subchronic toxicity after 4-week E. longifoliapowdered root treatment. Urinlysis of subchronic toxicity after 13-week E. longifolia powdered root treatment. [file 102987.f1.pdf]

Supplementary Table 1. The cytotoxicity evaluation (MTT assay) on *E. longifolia* powdered root treated CHO-K1 cells.

E. longifolia **powdered root Conc. (mg/mL)**	**Cell Viability (%)**
0	100.00
0.3125	106.54
0.625	107.89
1.25	109.27
2.5	122.78
5	93.11

1. The values were presented as Mean ± S.D. (N≥3).
2. Solvent (Sterile water) was used as negative control.
3. CHO-K1 cell was incubated with test compounds for 24 h, then the cytotoxic effects were evaluated by MTT assay. No cytotoxic effects were observed at any concentrations of test compound treatments.

Supplementary Table 2. The record of 14-day acute toxicity study of *E. longifolia* powdered root.

Supplementary Table 2-1. Mean Body Weight

	Treatment (g/kg b.w.)									
	Male					Female				
	0	1	2	4	6	0	1	2	4	6
**Day 0**	227.0±1.9	230.3±1.1	235.8±1.8	233.0±2.8	231.3±3.5	205.3±2.5	210.0±0.8	206.8±7.0	203.3±4.1	209.3±9.9
**Day 7**	273.8±1.4	277.3±3.7	283.0±3.3	280.8±1.9	280.8±4.6	221.0±5.2	220.3±3.7	220.5±9.6	218.5±3.1	221.8±13.8
**Day 14**	303.5±3.7	310.5±5.1	317.5±5.4	318.3±4.5	302.8±5.7	236.3±9.5	230.3±7.1	233.8±12.6	233.3±6.1	233.5±18.3

Supplementary Table 2-2. Hematology analysis

Items (Abbreviation)	Unit	Treatment (g/kg b.w.)									
		Male					Female				
		0	1	2	4	6	0	1	2	4	6
**Red blood cell count (RBC)**	10^6/μL	6.90±0.76	7.09±0.11	7.18±0.23	7.14±0.09	7.35±0.15	7.40±0.29	7.24±0.24	7.28±0.32	7.40±0.07	7.40±0.29
**Hematocrit (HCT)**	%	42.3±4.6	43.4±1.0	43.8±1.4	42.6±0.3	44.6±0.4	41.3±1.4	40.7±1.3	39.8±0.7	41.3±1.3	41.3±1.4
**Hemoglobin (Hb)**	g/dL	13.2±1.4	13.8±0.3	14.1±0.4	13.8±0.1	14.3±0.2	13.3±1.7	14.1±0.2	13.6±0.2	12.9±0.6	13.3±1.7
**Mean corpuscular hemoglobin (MCH)**	pg	19.2±0.2	19.5±0.1	19.7±0.1	19.4±0.3	19.5±0.6	18.0±1.8	19.5±0.9	18.7±0.7	19.6±0.5	18.0±1.8
**MCH concentration (MCHC)**	g/dL	31.2±0.5	31.8±0.5	32.3±0.2	32.4±0.2	32.1±0.2	32.2±3.5	34.7±1.4	34.1±0.4	31.8±0.8	32.2±3.5
**Mean corpuscular volume (MCV)**	fL	61.4±0.4	61.3±1.48	61.0±0.2	59.7±1.2	60.7±1.6	55.9±1.4	56.3±0.6	54.8±2.5	59.5±3.3	55.9±1.4
**White blood cell count (WBC)**	10^3/μL	5.43±1.45	5.03±0.08	4.33±0.34	4.33±0.65	4.85±0.71	1.45±0.85	1.31±0.35	2.23±0.87	1.84±0.58	1.45±0.85
**Lymphocytes**	%	88.0±1.1	86.8±0.9	88.0±0.8	85.3±2.3	87.8±1.9	61.7±3.9	66.3±8.3	74.9±6.3	75.5±8.5	61.7±3.9
**Neutrophils**	%	10.0±1.1	12.8±0.9	11.5±0.7	14.3±0.5	11.5±1.9	31.1±6.3	22.5±3.3	22.5±6.2	19.5±9.8	31.1±6.3
**Monocytes**	%	2.00±0.00	0.50±0.29	0.50±0.29	0.50±0.29	0.75±0.48	2.83±4.28	8.55±8.04	1.18±0.43	3.70±2.66	2.83±4.28
**Eosinophils**	%	0.00±0.00	0.00±0.00	0.00±0.00	0.00±0.00	0.00±0.00	2.90±1.99	2.58±1.65	1.50±0.56	0.90±0.18	2.90±1.99
**Basophils**	%	0.00±0.00	0.00±0.00	0.00±0.00	0.00±0.00	0.00±0.00	1.50±2.74	0.15±0.30	0.00±0.00	0.40±0.67	1.50±2.74
**Platelets count (PLT)**	10^3/μL	837.3±186.4	1103.8±127	1059.5±48.3	1280.5±44.2	1411.0±34.5	665.5±61.7	729.3±65.5	725.3±43.8	703.8±41.6	635.8±25.0
**Red cell distribution width (RDW)**	fL	13.7±0.4	19.0±3.9	12.9±0.3	13.2±0.4	14.1±0.6	13.1±0.5	12.5±0.6	12.6±0.2	12.4±0.3	12.6±0.3
**Pletelet Distribution Width (PDW)**	fL	25.7±7.1	28.9±6.0	31.2±0.4	31.6±0.9	32.5±0.7	8.48±0.13	8.78±0.33	8.75±0.24	8.13±0.29	8.48±0.43
**Mean platelet volume (MPV)**	fL	7.60±0.17	7.73±0.13	7.80±0.20	7.88±0.24	7.83±0.19	7.95±0.13	8.08±0.10	8.13±0.17	7.73±0.15	7.93±0.17

Supplementary Table 2-3. Serum biochemistry analysis

Items (Abbreviation)	Unit	Treatment (g/kg b.w.)									
		Male					Female				
		0	1	2	4	6	0	1	2	4	6
**Calcium**	**mg/dL**	11.5±0.8	9.9±0.4	9.7±0.4	9.2±0.3	8.7±0.7	13.1±0.9	12.7±1.3	11.5±0.5	13.2±1.4	12.7±2.0
**Chloride**	**mmol/L**	7.70±0.46	6.45±0.37	6.38±0.42	5.68±0.80	5.45±0.68	106.3±14.0	99.8±1.5	96.5±5.8	99.0±5.5	100.5±13.8
**Potassium**	**mmol/L**	6.10±0.80	5.18±0.24	5.05±0.29	5.03±0.36	4.85±0.26	8.35±1.17	8.43±0.50	8.55±0.64	8.65±3.88	9.55±2.07
**Sodium**	**mmol/L**	170.5±12.0	154.3±10.8	163.0±9.8	146.5±2.0	166.0±9.8	142.3±13.9	133.3±1.3	130.0±8.0	132.5±10.4	135.5±15.8
**Magnesium**	**mEg/L**	4.71±0.18	3.61±0.28	3.55±0.28	3.47±0.49	3.20±0.28	2.16±0.62	1.57±0.13	1.38±0.36	2.00±0.36	1.64±0.38
**Glucose**	**mg/dL**	95.8±26.0	94.3±6.6	99.5±11.6	75.5±11.4	81.0±3.3	90.5±11.8	87.5±7.9	92.3±2.1	98.0±35.3	98.8±14.9
**Alanine aminotransferase (ALT)**	**U/L**	22.5±1.2	22.0±2.3	21.3±3.4	20.5±1.6	17.3±1.3	23.8±4.0	22.0±0.0	18.5±2.9	25.3±2.5	27.8±3.3
**Aspartate aminotransferase (AST)**	**U/L**	140.0±12.3	108.5±11.9	101.5±17.6	90.8±4.7	84.5±14.0	150.0±38.0	137.3±12.5	107.0±23.9	131.5±48.5	115.5±33.8
**Alkaline phosphatase (ALP)**	**U/L**	116.5±26.6	110.8±15.2	112.5±23.2	94.3±9.95	107.0±12.4	54.8±18.2	47.3±9.5	50.5±10.3	54.3±7.1	50.0±9.8
**Creatinine**	**mg/dL**	0.88±0.09	0.78±0.05	0.75±0.03	0.65±0.09	0.60±0.08	0.73±0.05	0.70±0.00	0.70±0.08	0.88±0.17	0.75±0.10
**Blood urea nitrogen (BUN)**	**mg/dL**	24.3±1.4	22.3±2.7	22.0±1.8	19.8±3.2	19.3±4.1	28.0±2.8	26.8±1.5	25.3±4.2	30.0±6.1	32.0±6.8
**Albumin**	**g/dL**	4.20±0.29	3.78±0.19	3.60±0.26	3.23±0.48	3.05±0.44	3.93±0.50	3.78±0.25	3.38±0.29	3.98±0.10	3.60±0.41
**Globulin**	**U/L**	3.50±0.19	2.68±0.18	2.78±0.16	2.45±0.32	2.40±0.25	2.68±0.31	2.48±0.96	2.50±0.82	2.78±0.19	2.58±0401
**Total protein**	**g/dL**	7.70±0.46	6.45±0.37	6.38±0.42	5.68±0.80	5.45±0.69	6.60±0.81	6.25±0.17	5.88±0.26	6.68±0.28	6.18±0.68
**Cholesterol**	**mg/dL**	97.3±14.8	77.9±3.3	82.6±11.1	80.6±12.0	70.1±10.1	74.0±11.5	70.0±15.6	67.9±6.3	79.1±5.5	75.5±14.3
**Triglycerides**	**mg/dL**	61.0±8.8	42.8±1. 9	56.3±10.4	39.8±4.8	39.0±5.9	49.3±14.1	56.5±6.2	38.5±6.6	54.0±16.3	45.0±11.2
**Creatine phosphate kinase (CPK)**	**U/L**	541.8±91.8	476.8±130.8	281.5±42.0	206.0±41.4	170.8±52.7	625.3±349.5	535.0±46.4	292.8±73.0	245.3±154.6	251.3±112.5
**Lactate dehydrogenase (LDH)**	**U/L**	921.8±236.2	629.5±104.4	472.3±87.9	317.3±87.8	284.3±100.6	1056.0±414.3	1024.8±112.6	693.0±267.6	506.3±464.2	601.3±302.5
**Amylase**	**U/L**	840.0±158.3	822.5±48.9	723.5±78.0	681.5±88.1	643.0±69.3	407.8±58.9	384.8±67.6	397.8±112.8	528.0±158.4	307.8±17.3

Body weight in each of the treated group increased accordingly to the normal growth curve. Hematology and serum biochemistry analysis were all normal and unaffected by the *E. longifolia* powdered root ingestions. Data were expressed as Mean ± S.D. (N = 4). Statistical analysis: * $p < 0.05$, ** $p < 0.01$, and *** $p < 0.001$, indicate a statistical difference with the control group by One-way ANOVA. Rats in control group were received single gavage of vehicle (sterile water).

Supplementary Table 3. The record of mean body weight in 13-week subchronic toxicity study (*E. longifolia* powdered root)

	Treatment (g/kg b.w.)							
	Male				Female			
	0	0.6	1.2	2	0	0.6	1.2	2
**Week 0**	219.5±7.3	222.0±8.3	226.1±5.6	225.0±10.0	219.6±8.7	216.4±16.3	219.2±11.6	218.8±9.8
**Week 1**	284.3±12.3	285.8±7.4	285.6±14.4	279.6±12.0	238.7±11.9	232.8±19.6	230.7±17.2	235.9±12.0
**Week 2**	324.1±19.1	319.9±11.2	315.3±20.9	314.8±16.0	250.3±14.2	246.2±20.4	248.5±18.5	249.9±18.3
**Week 3**	350.5±23.2	354.4±12.4	353.5±24.2	346.6±23.2	264.8±15.4	255.1±21.5	256.1±22.8	256.4±13.8
**Week 4**	380.8±29.2	371.6±14.7	367.0±28.1	372.2±29.6	276.2±13.7	256.4±19.8	265.9±21.7	264.0±13.3
**Week 5**	399.0±31.6	391.6±15.6	396.8±29.3	389.3±33.2	280.3±17.2	269.6±22.6	273.5±26.9	269.8±12.5
**Week 6**	393.2±48.9	416.0±20.0	411.7±34.0	399.8±31.6	281.5±20.6	268.7±24.8	277.0±26.0	277.6±15.0
**Week 7**	433.4±37.8	430.6±21.1	431.8±34.2	422.7±37.3	275.3±29.0	272.1±32.6	278.2±23.3	275.2±13.1
**Week 8**	445.2±38.6	442.7±22.5	437.4±35.1	434.4±38.3	297.5±20.4	289.6±24.3	284.6±27.1	289.4±17.9
**Week 9**	472.2±39.5	458.2±24.1	452.5±38.5	452.7±39.3	294.5±19.5	283.3±27.1	287.0±30.9	283.9±12.6
**Week 10**	473.3±45.0	456.7±25.4	453.3±50.5	430.9±72.2	296.7±20.4	300.5±45.9	292.5±33.6	294.9±21.3
**Week 11**	473.1±46.8	470.0±25.7	464.6±50.3	470.5±49.6	300.3±19.8	293.5±28.2	289.8±32.8	292.0±17.4
**Week 12**	492.0±44.9	476.6±26.1	469.6±47.9	461.1±52.0	308.1±21.1	293.4±27.6	292.9±32.2	290.4±15.9
**Week 13**	503.1±45.1	486.6±26.5	473.9±50.8	465.2±49.7	297.1±20.0	284.8±29.7	282.4±26.5	303.8±21.5

Body weight in each of the treated group increased accordingly to the normal growth curve. Data were expressed as Mean ± S.D. (N = 10). Statistical analysis: * $p < 0.05$, ** $p < 0.01$, and *** $p < 0.001$, indicate a statistical difference with the control group by One-way ANOVA. Rats in control group were received single gavage of vehicle (sterile water).

Supplementary Table 4. Hematology and serum biochemistry profiles of subacute toxicity after 4-week *E. longifolia* powdered root treatment.

Supplementary Table 4-1. Hematology analysis

Items (Abbreviation)	Unit	Treatment (g/kg b.w.)			
		Male			
		0	0.6	1.2	2
Red blood cell count (RBC)	10^6/μL	7.49±0.21	7.38±0.22	7.55±0.26	7.64±0.32
Hematocrit (HCT)	%	14.3±0.5	14.1±0.4	14.4±0.9	14.6±0.5
Hemoglobin (Hb)	g/dL	44.1±1.6	43.2±1.4	43.9±2.0	43.4±1.4
Mean corpuscular hemoglobin (MCH)	pg	19.1±0.5	19.2±0.4	19.2±1.3	19.2±0.8
MCH concentration (MCHC)	g/dL	32.5±0.6	32.7±0.5	32.7±1.8	33.7±0.7
Mean corpuscular volume (MCV)	fL	58.9±1.2	58.6±1.5	58.2±1.9	57.0±1.7
Red cell distribution width (RDW)	fL	12.6±1.4	30.3±1.1	30.1±1.0	29.5±0.6
Mean platelet volume (MPV)	fL	7.96±0.31	8.03±0.38	7.99±0.31	8.10±0.35
Pletelet Distribution Width (PDW)	fL	30.3±0.7	12.7±0.9	12.8±1.0	12.9±0.8
White blood cell count (WBC)	10^3/μL	7.50±2.59	6.67±1.27	6.70±1.29	8.33±2.69
Lymphocytes	10^3/mm^3	83.3±2.7	85.5±2.1	85.3±3.6	83.7±3.1
Neutrophils	10^3/mm^3	15.6±2.6	13.8±2.3	14.5±3.6	15.6±3.3
Monocytes	10^3/mm^3	1.10±0.57	0.70±0.67	0.20±0.42*	0.70±0.67
Eosinophil	10^3/mm^3	0.00±0.00	0.00±0.00	0.00±0.00	0.00±0.00
Basophils	10^3/mm^3	0.00±0.00	0.00±0.00	0.00±0.00	0.00±0.00
Platelets count (PLT)	10^3/μL	1010±344	1065±80	1110±168	1167±205
Prothrombin time (PT)	sec	11.5±1.3	10.7±0.4	10.5±0.2	10.4±0.2*
Partial thromboplastin time (APTT)	sec	29.9±7.1	24.5±2.9	24.6±2.7	22.8±2.0*
Fibrinogen (Fbg)	mg/dL	200.8±34.3	204.6±10.4	205.9±16.8	238.4±33.3

Hematology profiles were within normal range (Charles River, 2008) and unaffected by the *E. longifolia* powdered root ingestions. Data were expressed as Mean ± S.D. (N = 8-10).

Supplementary Table 4-2. Serum biochemistry analysis

Items (Abbreviation)	Units	Treatment (g/kg b.w.)			
		Male			
		0	0.6	1.2	2
**Calcium**	**mg/dL**	10.6±0.5	10.8±1.4	9.7±0.8	10.1±0.9
**Chloride**	**mmol/L**	109.1±5.8	117.8±13.4	103.5±4.3	102.1±4.1
**Phosphorus**	**mg/dL**	9.14±0.81	9.10±1.26	8.16±0.56	9.65±1.78
**Potassium**	**mmol/L**	5.00±0.45	4.94±0.45	4.72±0.40	4.76±0.82
**Sodium**	**mmol/L**	147.8±7.2	157.5±17.5	139.1±6.8	139.2±5.5
**Magnesium**	**mEg/L**	3.13±0.20	3.11±0.31	3.14±0.22	3.26±0.61
**Glucose**	**mg/dL**	121.3±21.7	140.2±31.3	122.3±20.1	163.3±48.7
**Alanine aminotransferase (ALT)**	**U/L**	20.7±3.3	20.3±3.7	19.9±3.5	23.1±4.3
**Aspartate aminotransferase (AST)**	**U/L**	93.4±9.0	82.8±14.9	77.8±12.4*	73.3±11.4*
**Alkaline phosphatase (ALP)**	**U/L**	84.7±27.2	80.7±15.4	73.3±13.4	73.5±9.3
**Gamma glutamyl transpeptidase (GGT)**	**mg/dL**	0.98±0.19	0.82±0.15	0.92±0.40	1.37±0.76
**Creatinine**	**mg/dL**	0.80±0.07	0.82±0.11	0.71±0.06	0.75±0.08
**Blood urea nitrogen (BUN)**	**mg/dL**	24.1±2.7	20.7±3.7*	17.5±1.5*	17.2±3.1*
**Albumin**	**g/dL**	3.67±0.22	3.97±0.48	3.53±0.35	3.66±0.33
**Globulin**	**U/L**	2.89±0.36	2.81±0.42	2.57±0.42	2.74±0.42
**Total protein**	**g/dL**	6.56±0.51	6.78±0.86	6.10±0.71	6.40±0.66
**Cholesterol**	**mg/dL**	77.3±10.2	71.8±11.9	65.9±10.5*	62.6±20.5*
**Triglycerides**	**mg/dL**	45.0±7.2	41.3±8.0	47.8±11.9	45.9±9.9
**Creatine phosphate kinase (CPK)**	**U/L**	364.5±159.5	255.3±105.2	184.2±60.5*	140.1±40.3*
**Lactate dehydrogenase (LDH)**	**U/L**	525.1±130.1	388.8±114.6	295.7±92.0*	287.7±94.0*
**Amylase**	**U/L**	792.1±97.1	975.7±339.1	817.3±125.6	806.3±145.5

Most serum biochemical parameters were within normal range (Charles River, 2008), except of the pharmacological effects in AST, creatinine, BUN, CPK, LDH and chlosterol in treated males, after 13-week *E. longifolia* powdered root ingestions. Data were expressed as Mean ± S.D. (N

= 8-10). Statistical analysis: * $p < 0.05$, ** $p < 0.01$, and *** $p < 0.001$, indicate a statistical difference with the control group by One-way ANOVA. Rats in control group were received single gavage of vehicle (sterile water).

Supplementary Table 5. The absolute and relative tissue weights of subchronic toxicity after 4-week *E. longifolia* powdered root treatment.

		Treatment (g/kg b.w.)						Treatment (g/kg b.w.)			
		Male						Male			
		**0**	**0.6**	**1.2**	**2**			**0**	**0.6**	**1.2**	**2**
**ADRENALS**						**SPLEEN**					
Absolute Weight	gram	0.03±0.00	0.03±0.00	0.03±0.00	0.03±0.00	Absolute Weight	gram	0.79±.10	0.84±0.08	0.78±0.10	0.70±0.11
Ratio Per Body Weight	(10^{-3})	0.09±0.01	0.09±0.01	0.08±0.02	0.10±0.02	Ratio Per Body Weight	(10^{-3})	2.44±0.33	2.51±0.28	2.38±0.34	2.38±0.32
**HEART**						**TESTES**					
Absolute Weight	gram	0.98±0.09	1.06±0.07	1.04±0.08	0.94±0.14	Absolute Weight	gram	1.60±0.16	1.59±0.18	1.62±0.14	1.49±0.18
Ratio Per Body Weight	(10^{-3})	3.03±0.27	3.16±0.20	3.17±0.22	3.18±0.31	Ratio Per Body Weight	(10^{-3})	4.94±0.47	4.74±0.52	4.95±0.56	5.08±0.65
**KIDNEYS**						**THYMUS**					
Absolute Weight	gram	0.97±0.08	1.04±0.08	1.05±0.05	0.95±0.11	Absolute Weight	gram	0.44±0.10	0.45±0.05	0.48±0.13	0.36±0.12
Ratio Per Body Weight	(10^{-3})	2.99±0.18	3.10±0.19	3.20±0.14	3.22±0.23	Ratio Per Body Weight	(10^{-3})	1.37±0.33	1.34±0.13	1.47±0.44	1.20±0.34
**LIVER**						**LUNG**					
Absolute Weight	gram	8.55±0.63	8.76±0.61	8.66±0.78	8.27±1.74	Absolute Weight	gram	1.55±0.24	1.60±0.34	1.43±0.19	1.48±0.29
Ratio Per Body Weight	(10^{-3})	26.5±2.6	26.1±1.4	26.4±1.7	27.9±4.1	Ratio Per Body Weight	(10^{-3})	4.79±0.76	4.78±1.12	4.37±0.64	5.07±1.21

After the 4-week treatment, the wet organ weights, and related organ weight to body weight remained unaffected in compared to control group. Data were expressed as Mean ± S.D. (N = 10). Statistical analysis: * $p < 0.05$, ** $p < 0.01$, and *** $p < 0.001$, indicate a statistical difference with the control group by One-way ANOVA.

Supplementary Table 6. Urinlysis of subchronic toxicity after 13-week *E. longifolia* powdered root treatment.

		Treatment (g/kg b.w.)							
		Male				Female			
		**0**	**0.6**	**1.2**	**2**	**0**	**0.6**	**1.2**	**2**
**Volume**	**mL**	24.3±4.4	24.6±2.6	18.0±5.4	15.4±3.3	12.6±3.5	13.6±3.3	12.1±4.7	12.4±3.1
**S. Gravity**		1.02±0.00	1.02±0.00	1.02±0.00	1.02±0.00	1.03±0.00	1.02±0.00	1.02±0.00	1.02±0.00
**pH**		9.00±0.00	9.00±0.00	8.60±0.52	8.60±0.52	8.90±0.32	8.80±0.63	8.10±0.57	8.90±0.32
**Urobilirubin**		0.10±0.00	0.10±0.00	0.10±0.00	0.10±0.00	0.10±0.00	0.10±0.00	0.10±0.00	0.10±0.00

Data were expressed as Mean ± S.D. (N = 8-10). Statistical analysis: * $p < 0.05$, ** $p < 0.01$, and *** $p < 0.001$, indicate a statistical difference with the control group by One-way ANOVA.

Histopathological Report

13-week Subchronic Oral Toxicity of Tongkat Ali in Rats

Materials and Methods:

This study was entrusted by the School of Pharmacy, Taipei Medical University to assess the pathological changes of Tongkat Ali in the 13-week subchonic oral toxicity in rats. Rats (Wistar strain), 10 males and 10 females in each group, were daily gavaged with Tongkat Ali product at dose of 0 g/kg (control), 0.6 g/kg (low dose), 1.2 g/kg (middle dose), and 2 g/kg (high dose) body weight for 13-week.

Representative tissues including: adrenals, aorta, bone marrow and femur, brain, spinal cord, esophagus, eye, heart, duodenum, jejunum, ileum, cecum, colon, rectum, kidney, liver, lung, lymph node, spleen, ovary (female), or testes (male), spleen, pancreas, prostate gland, sciatic nerve, seminal vesicle, stomach, skeletal muscle, thymus, urinary bladder, uterus, vagina (female) in the control and high dose groups were fixed in 10% neutral buffered formalin for pathologic examination (Pathological Table 1). Tissues were further processed, embedded in paraffin, cut at 2 μm by microtone (Leica RM 2145, Nussloch, Germany), and stained with Hematoxylin & Eosin (H&E) staining and evaluated under light microscope (BX-51, Olympus, Tokyo, Japan) for histopathological evaluation. The pathological nomenclatures in each organ were listed in Pathological Table 2. Severity of lesions was

graded. Degree of lesions was graded from one to five depending on severity: 1 = minimal (< 1%); 2: slight (1-25%); 3 = moderate (26-50%); 4 =moderate/severe (51-75%); 5 = severe/high (76-100%) (Pathological Table 2).

Results:

Gross findings: No significant lesions of adrenals, heart, kidney, liver, lung, spleen, thymus and testes (male rats) and ovary (female rats) (Pathological Figure 1 & 2), and other organs were found in the control and high dose treated groups.

Histopathological findings: No significant lesions of adrenals, aorta, bone marrow and femur, brain, spinal cord, esophagus, eye, heart, duodenum, jejunum, ileum, cecum, colon, rectum, kidney, liver, lung, lymph node, spleen, ovary (female), or testes (male), spleen, pancreas, prostate gland, sciatic nerve, seminal vesicle, stomach, skeletal muscle, thymus, urinary bladder, uterus, vagina (female) were found in between the control and high dose treated groups.

Nonspecific findings:

1. Adrenal gland showed extensive to moderate coagulative necrosis and calcification (Pathological Figure 5A). The incidence of female rat in the high dose group was 1/10.
2. Hearts showed focal, slight mononuclear cell infiltration (Pathological Figure 5B). The incidence was 2/10 in male rats and 1/10 in female rats of control group, respectively.

3. Kidneys showed focal, minimal to slight tubular cyst formation (Pathological Figure 5C). The incidence was 1/10 in male and female rats of high dose group.
4. Prostate glands showed focal, minimal to slight mononuclear cell infiltration in the interstitial area (Pathological Figure 5D). The incidence was 2/10 in male rats of control and high dose groups.
5. Cervix showed focal, slight glandular cyst (Pathological Figure 5E). The incidence was 1/10 in female rats of control group. Uterus showed bilateral, slight to moderate severe tubular dilation (Pathological Figure 5F). The incidence was 1/10 and 3/10 in female rats of control and high dose groups, respectively. In addition, focal glandular cyst of cervix, and bilateral luminal dilation of uterus presented in female rats of control and high dose groups that were considered as a physical change during estrus cycle of normal female rats.
6. The incidence and grade of lesion in adrenal gland, heart, kidney, prostate glands, cervix, and uterus were not considered substance related between the control and high dose groups.

Conclusion

From the histopathological examination, no significant toxics or dose-related effects of this compound to organs were observed under the microscopic examination in the control and high dose groups.

Pathological Figure 1

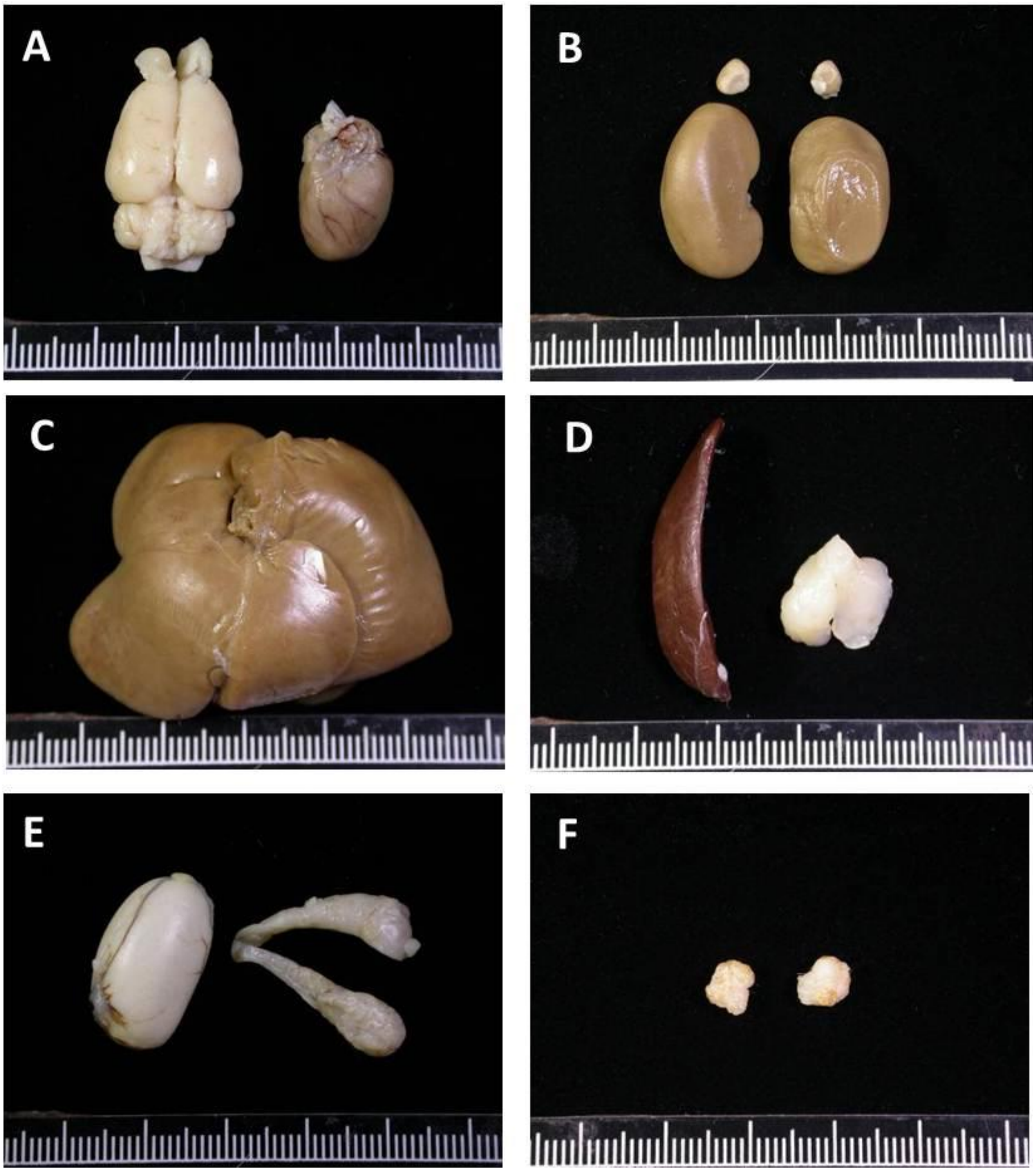

Pathological Figure 1. Gross findings of the control rats in the Tongkat Ali 13-week subchronic oral toxicity test. No significant gross findings of brain and heart (A), adrenal and kidney (B), liver (C), spleen and thymus (D), testes and epididymis (E) (animal no.: M101), and ovary (F) (animal no.: F101) were noted in the control rats.

Pathological Figure 2

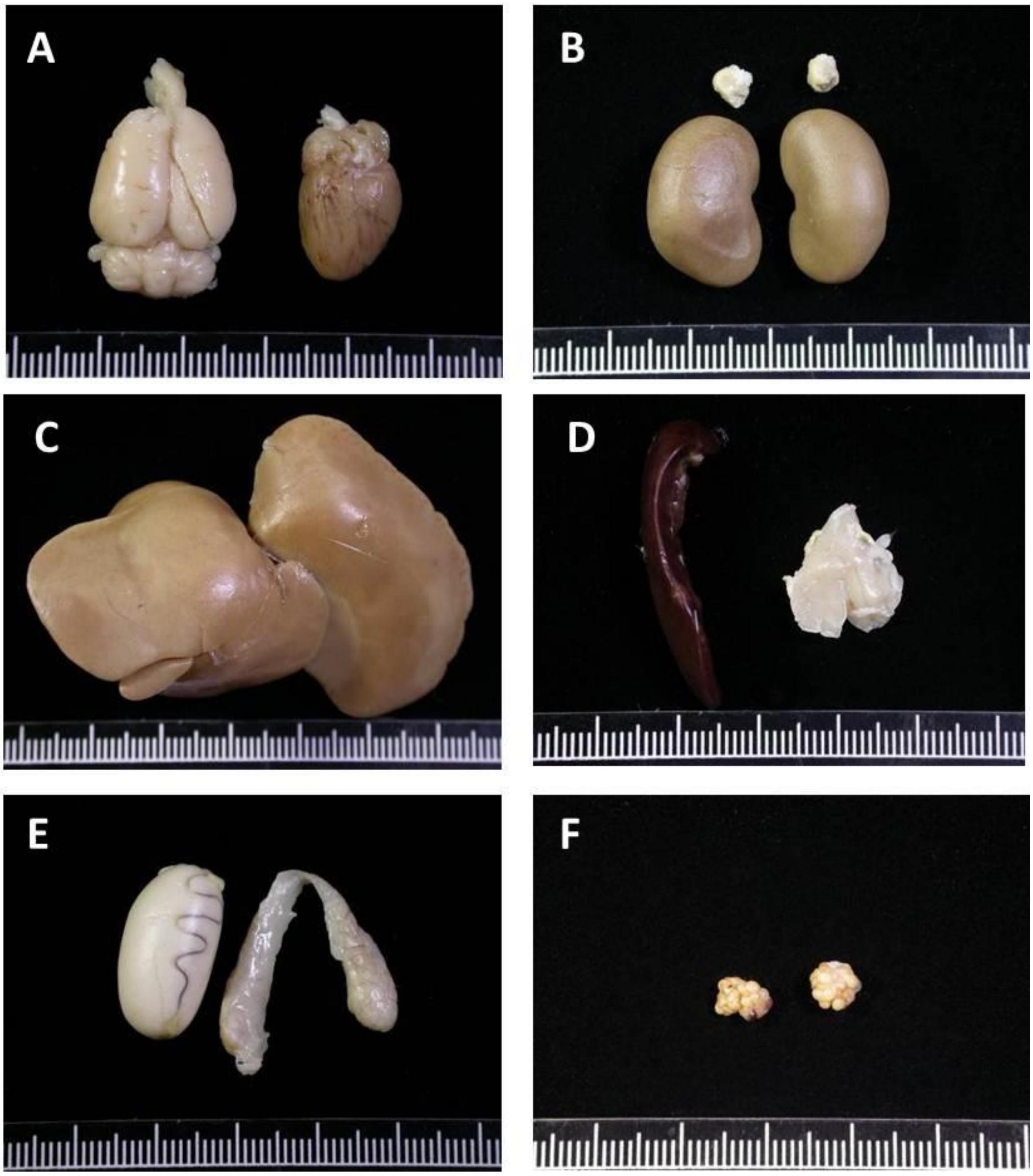

Pathological Figure 2. Gross findings of the high dose-treated rats in the Tongkat Ali 13-week subchronic oral toxicity test. No significant gross findings of brain and heart (A), adrenal and kidney (B), liver (C), spleen and thymus (D), testes and epididymis (E) (animal no.: M231), and ovary (F) (animal no.: F231) were noted in the high dose-treated rats.

Pathological Figure 3

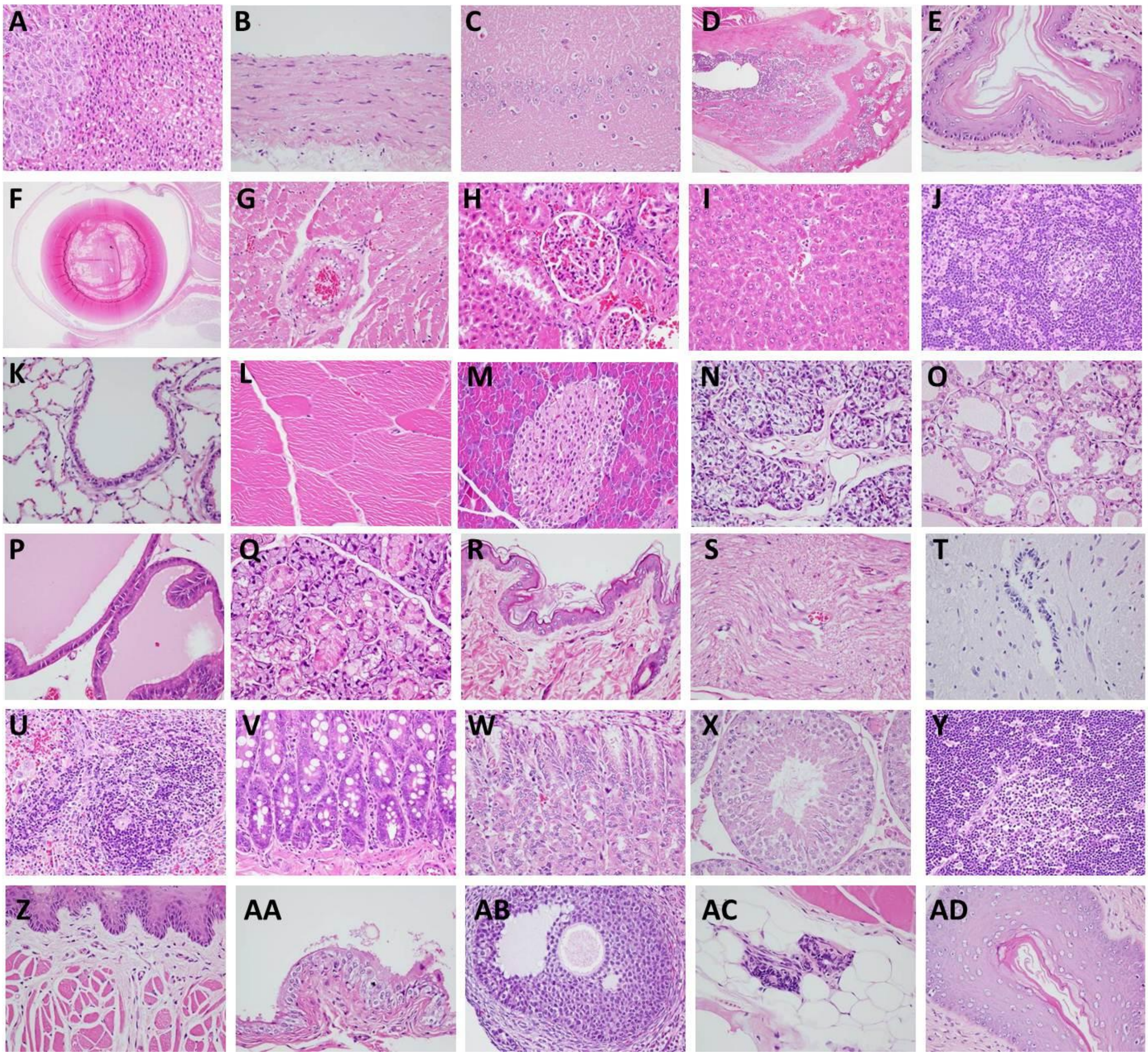

Pathological Figure 3. Histopathological changes of rats in the control group. No significant histopathologic changes in the adrenal (A), aorta (B), brain (C), femur (D), esophagus (E), eye (F), heart (G), kidney (H), liver (I), lymph node (J), lung (K), muscle (L), pancreas (M), parathyroid (N), thyroid (O) (animal code: M101), prostate (P), salivary gland (Q), skin (R), sciatic nerve (S), spinal cord (T), spleen (U), small intestine (V), stomach (W), testes (X), thymus (Y), tongue (Z), urinary bladder (AA) (animal code: M101), ovary (AB), mammary gland (AC), and vagina (AD) (animal code: F101), H&E.

Pathological Figure 4

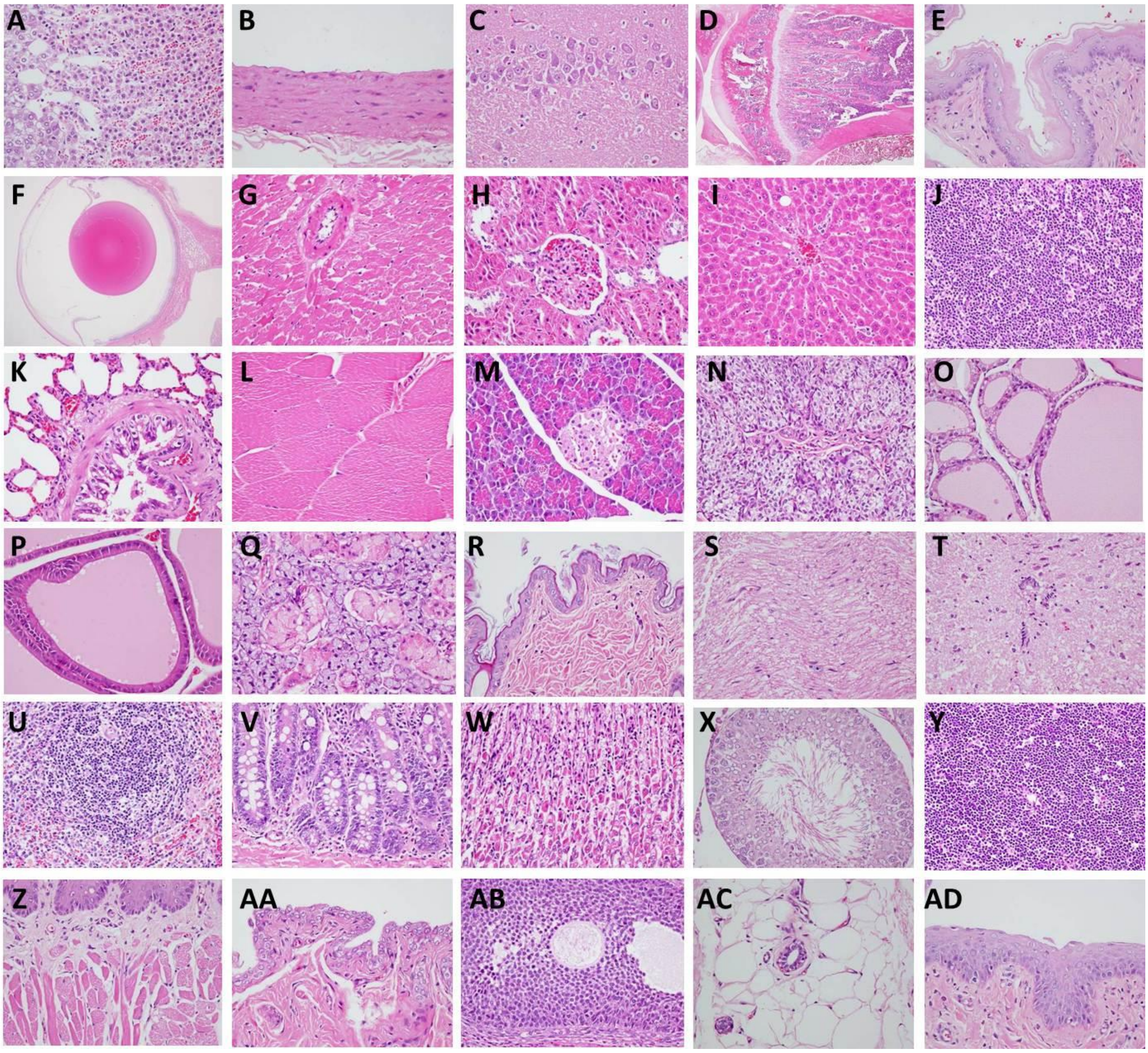

Pathological Figure 4. Histopathological changes of rats in the high dose-treated group. No significant histopathologic changes in the adrenal (A), aorta (B), brain (C), femur (D), esophagus (E), eye (F), heart (G), kidney (H), liver (I), lymph node (J), lung (K), muscle (L), pancreas (M), parathyroid (N), thyroid (O) (animal code: M132), prostate (P), salivary gland (Q), skin (R), sciatic nerve (S), spinal cord (T), spleen (U), small intestine (V), stomach (W), testes (X), thymus (Y), tongue (Z), urinary bladder (AA) (animal code: M132), ovary (AB), mammary gland (AC), and vagina (AD) (animal code: F133), H&E.

Pathological Figure 5

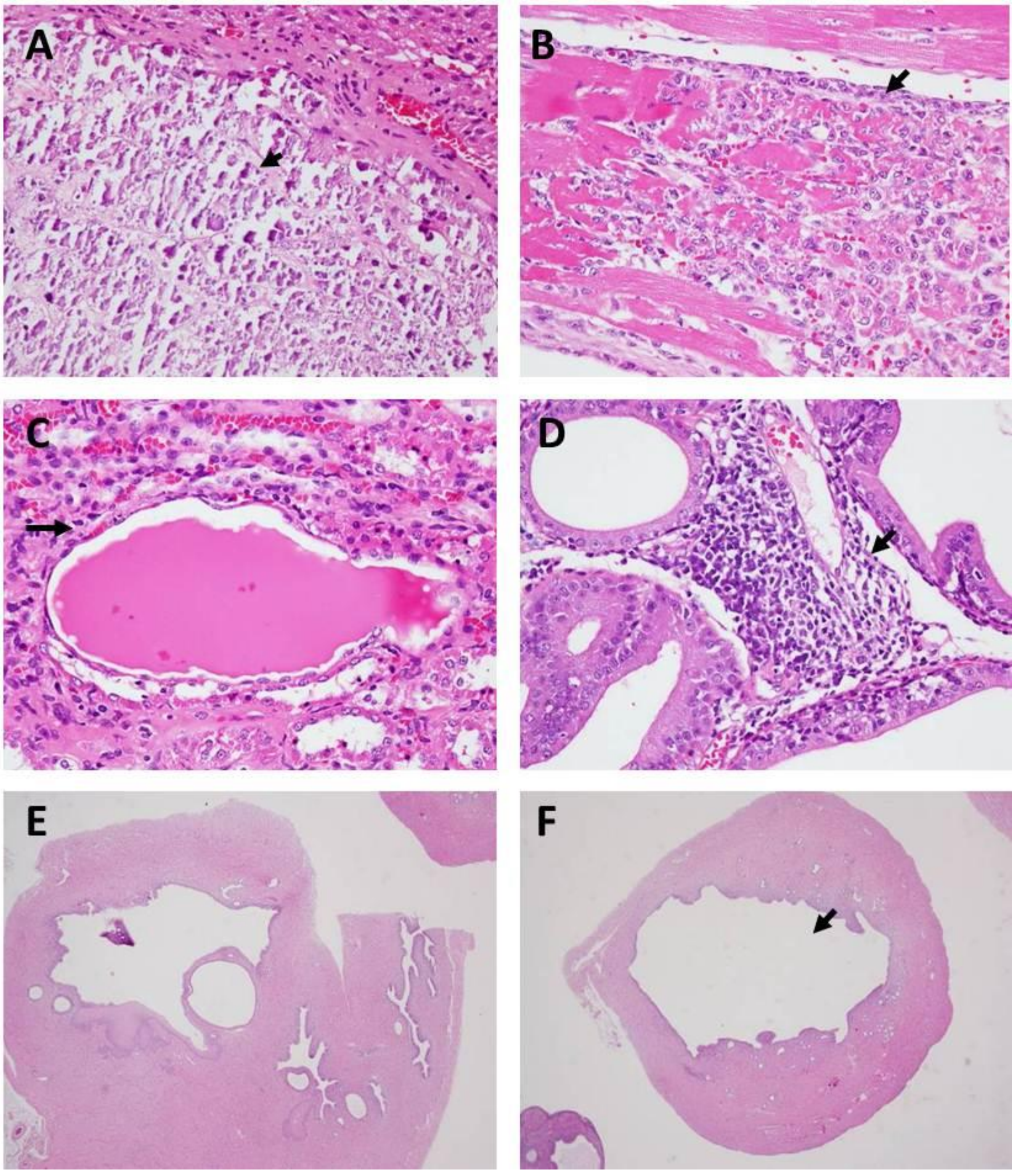

Pathological Figure 5. Nonspecific histopathological alterations of rats. Focal, moderate, coagulative necrosis and calcification of adrenal gland (A. 400x, animal code: F133); focal, slight, mononuclear cell infiltration of heart (B. 400x, animal code: M202); focal minimal hyaline cast of kidneys (C. 400x, animal code: M134); focal, slight, mononuclear cell infiltration of prostate gland (D. 400x, animal code: M201); focal, slight glandular cyst of cervix (E. 40x, animal code: F104) and focal, slight, luminal dilation of uterus (F. 40x, animal code: F 104) were randomly noted in the control and high dose-treated rats. H&E stain.
